# Ginsenoside Re Attenuates D-Galactose-Induced Aging-like Oxidative Stress in Cultured Bovine Oocytes via Activation of the SIRT1/Nrf2 Signaling Pathway

**DOI:** 10.3390/ijms27177569

**Published:** 2026-08-24

**Authors:** Seo-Young Hyeon, Chalani Dilshani Perera, Seung-Eun Lee, Ji-Hyo Kim, Sanghwa Yoon, Yang Jae Kang, Song Park, Muhammad Idrees, Il-Keun Kong

**Affiliations:** 1 Graduate School of Applied Life Science (BK21 Four), Gyeongsang National University, Jinju 52828, Gyeongnam, Republic of Korea; 2 Research Institute of Molecular Alchemy, Gyeongsang National University, Jinju 52828, Gyeongnam, Republic of Korea; 3 Division of Applied Life Science, Gyeongsang National University, Jinju 52828, Gyeongnam, Republic of Korea; 4 Division of Bio & Medical Bigdata Department (BK21 Four), Gyeongsang National University, Jinju 52828, Gyeongnam, Republic of Korea; 5 Division of Animal Science, Gyeongsang National University, Jinju 52828, Gyeongnam, Republic of Korea; 6 Institute of Agriculture and Life Science, Gyeongsang National University, Jinju 52828, Gyeongnam, Republic of Korea; 7 The King Kong Corp., Ltd., Gyeongsang National University, Jinju 52828, Gyeongnam, Republic of Korea

**Keywords:** Ginsenoside Re, oocyte aging, mitochondrial function, SIRT1/Nrf2 signaling, oxidative stress

## Abstract

Maternal aging is associated with increased oxidative stress and decreased mitochondrial function, which deteriorate oocyte quality. Ginsenoside Re (GRe), an antioxidant derived from *Panax ginseng*, has been reported to alleviate cellular damage and extend healthy lifespan. In this study, we investigated the protective effects of GRe on in vitro-matured bovine oocytes and elucidated the underlying mechanisms. Oocyte aging-like oxidative stress conditions were induced by adding 20 mg/mL D-galactose (D-gal) to the in vitro maturation (IVM) medium; GRe treatment involved supplementing the medium with various GRe concentrations (25, 50, and 100 μg/mL). GRe treatment significantly improved maturation, with 50 μg/mL GRe showing the most pronounced effect. This recovery was accompanied by reduced accumulation of reactive oxygen species and improved mitochondrial capacity and biogenesis, mediated by SIRT1/Nrf2 signaling. Consequently, GRe improved embryonic developmental ability. These findings suggest that GRe may improve oocyte quality in the context of aging and associated oxidative stress.

## 1. Introduction

Age-related infertility is increasing due to the increase in the average age of women giving birth to their first child, which is caused by several factors, such as changes in social awareness and higher education level [1]. In fact, the number of women giving birth to their first child after the age of 35 increased from 1/100 in 1970 to 1/12 in 2006 [2] and the proportion of women giving birth to their first child after the age of 30 increased sixfold between 1970 and 2002 and up to 2024 the average age of childbirth is approximately 34 [3,4]. Oocyte aging is accompanied by lipid peroxidation [5], chromosomal and spindle abnormalities [6], and telomere shortening [7]. Furthermore, the mitochondria, the most abundant organelle in oocytes, are critically affected by aging [8], including a reduction in mitochondrial DNA (mtDNA) copy number [9] and an increase in the frequency of mtDNA copy number mutations due to mitochondrial damage, resulting in decreased fertilization rates and abnormal embryo and fetal development [10].

The SIRT1 (Sirtuin 1)/Nrf2 (Nuclear factor erythroid 2-related factor 2) signaling pathway is involved in energy metabolism and aging [11]. Several studies have shown that SIRT1 plays a role in transcriptional regulation, chromatin modification, energy metabolism, and aging [12]. SIRT1 has been shown to deacetylate Nrf2, thereby increasing its stability and transcriptional activity and enhancing cell resistance to oxidative damage [13,14]. Nrf2 has been recognized as an important transcription factor that mediates protection against oxidants and enhances cell survival in many tissues. SIRT1 via Nrf2 plays a crucial role in female fertility by protecting oocytes from ROS and regulating antioxidant response [15,16]. Furthermore, sirtuins also inhibit ovarian fibrosis, which plays a critical role in activating the inflammasome (NLRP3) and nuclear factor κB (NF-κB) [17]. Aging significantly alters the SIRT1-Nrf2 pathway, which is critical for oocyte maturation and quality, as it protects the oocyte from oxidative stress and aging [18,19]. Several studies reported that activation of SIRT-1 significantly improves oocyte maturation rate and quality via boosting mitochondrial function [20].

Ginseng is a herbal medicine that has been widely used in the East for over 2000 years [21], and ginsenosides are one of the main physiologically active components of ginseng and include various types such as Rb1, Rb2, Rb3, Rc, Rd, Re, Rf, and Rg1 [22]. *Panax ginseng* extract and ginsenosides improve oocyte maturation, embryo quality, and embryo development [23,24]. Several ginsenosides have also been shown to modulate SIRT1 activity, thereby influencing aging-related cellular processes, including oxidative stress responses and mitochondrial homeostasis [25,26]. In addition, recent studies have demonstrated that GRe can activate the Nrf2 expression and enhance antioxidant defense mechanisms [26,27,28]. GRe has been attracting attention in recent years due to its diverse biological effects [28], including the enhancement of mitochondrial activity [29,30], anti-apoptotic properties [29], and protective effects against various forms of cellular damage [30].

However, the effect of GRe on the regulation of the SIRT1/Nrf2 signaling pathway remains unclear, more specifically in reproductive tissues. In this study, we evaluated the protective effects of GRe against oxidative stress-induced aging-like conditions during in vitro maturation of bovine oocytes. Bovine oocytes are widely used as a model for human oocyte maturation because they share key physiological features, including comparable cytoplasmic maturation processes, mitochondrial distribution patterns, and meiotic progression characteristics [31,32]. Furthermore, bovine oocytes naturally undergo age-related declines in mitochondrial function and redox homeostasis that parallel the physiological changes observed during human reproductive aging [33]. Collectively, these similarities suggest that bovine oocytes may be a reliable model for studying the aging process and evaluating the cellular and mitochondrial mechanisms underlying oocyte aging. Here, we investigated whether GRe exerts its effects by regulating mitochondrial function and activating the SIRT1/Nrf2 pathway. The results of this study provide mechanistic insights into the potential therapeutic use of GRe to improve oocyte quality under conditions of reproductive aging-like oxidative stress.

## 2. Results

### 2.1. Ginsenoside Re Restores In Vitro Oocyte Aging Induced by D-Galactose

**Figure 1:**
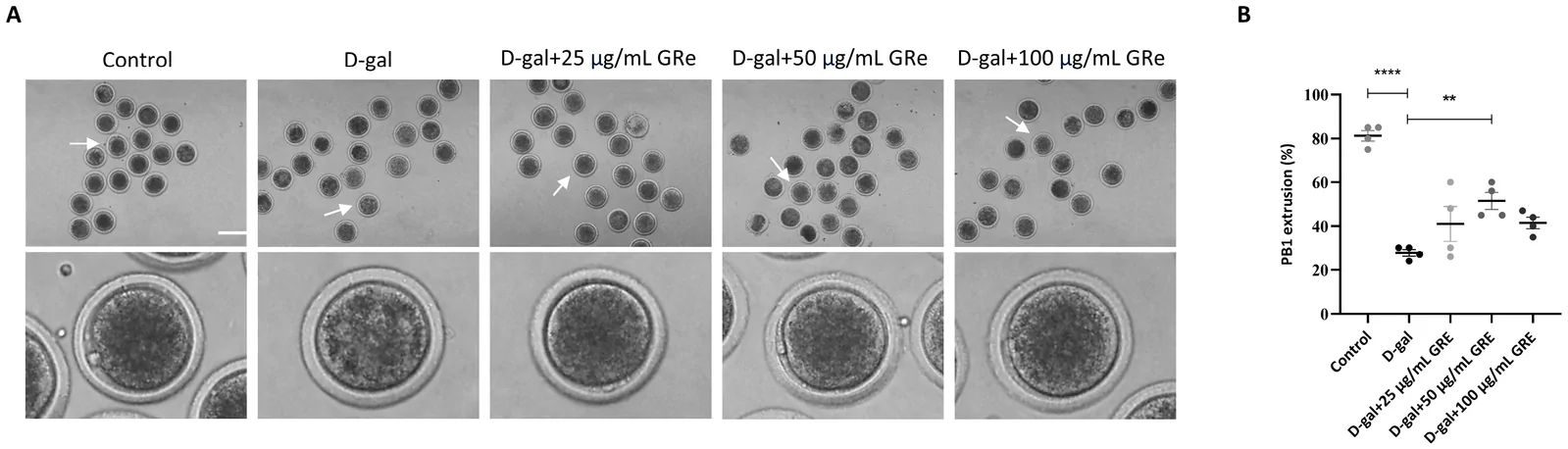
Effects of GRe treatment on oocyte maturation in aged oocytes. (**A**) Representative images of first polar body (PB1) extrusion in control, aged-like condition, and GRe-treated oocytes. Original magnification: ×64. (**B**) Percentage of PB1 extrusion (*n* = 80). The experiments were repeated four times, and the data are presented as mean ± SE. ** *p* < 0.01; **** *p* < 0.0001. D-gal (20 mg/mL D-gal); D-gal + GRe (20 mg/mL D-gal + 25 μg/mL); D-gal + GRe (20 mg/mL D-gal + 50 μg/mL); D-gal + GRe (20 mg/mL D-gal + 100 μg/mL); GRe; GRe, Ginsenoside Re.

We successfully established an in vitro aging-like oxidative stress model by supplementing IVM medium with 20 mg/mL D-gal. Induction of aging-like conditions significantly reduced the PB1 extrusion rate to 58 (55.24% ± 0.24) compared to 94 in the control group (84.45% ± 0.44) (*p* < 0.001) (Table S1). However, treatment with increasing concentrations of GRe (25, 50, and 100 µg/mL) resulted in a dose-dependent increase in maturation rate (Table S1). In particular, the PB1 extrusion rate was significantly improved in the group treated with 50 µg/mL GRe 90 (78.15% ± 0.26) (*p* < 0.01), indicating a restoration of oocyte maturation competence (Figure 1A,B). These results support the successful establishment of an aging-like oxidative stress model and demonstrate GRe’s potential to reverse oocyte aging. Additionally, the GRe-only treatment enhanced oocyte maturation rate shows the protective effect of GRe, acting directly on oocytes and neutralizing the adverse effects of D-galactose (Table S1).

### 2.2. Effects of GRe Against D-gal-Induced Oxidative Stress

**Figure 2:**
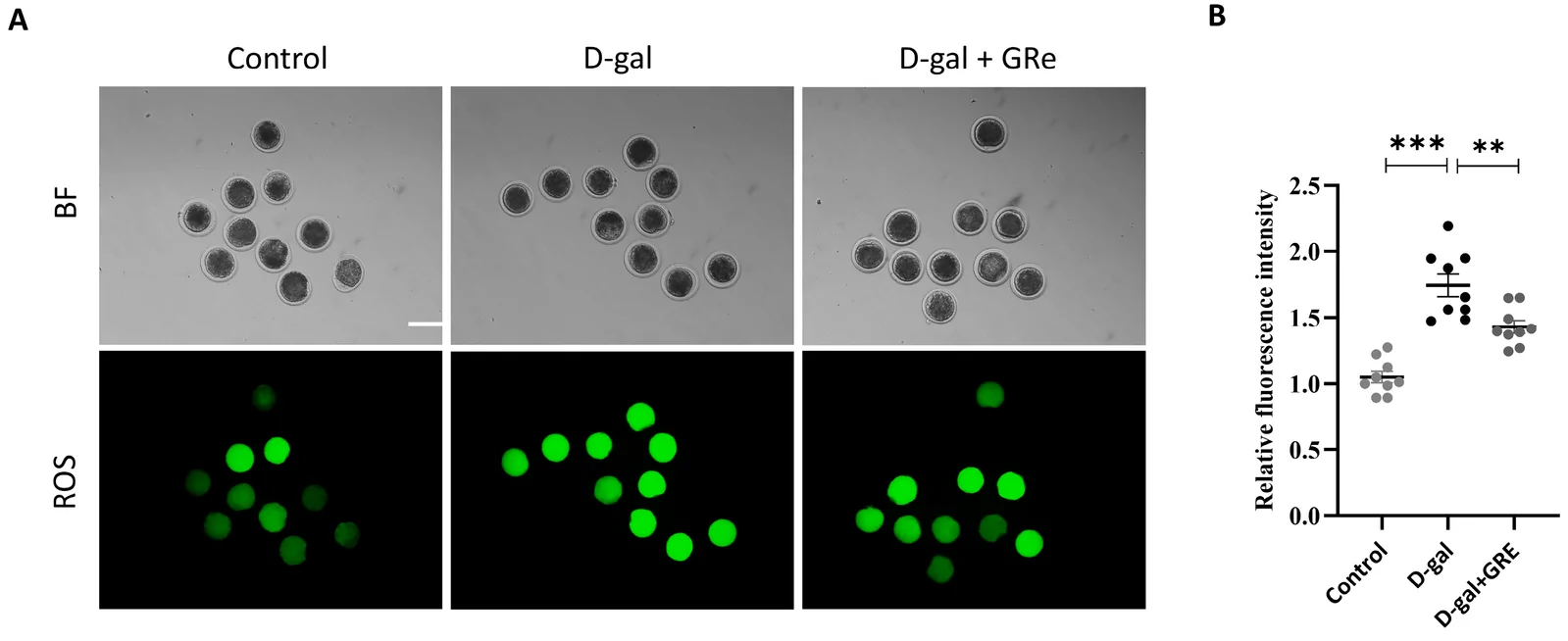
Effect of GRe treatment on intracellular ROS levels in aged oocytes. (**A**) Representative fluorescence images of ROS levels detected by H_2_DCFDA staining. Original magnification: ×100. (**B**) Quantification of ROS fluorescence intensity in each group (n = 30). The experiments were repeated three times, and the data are presented as mean ± SE. ** *p* < 0.01; *** *p* < 0.001. D-gal (20 mg/mL); D-gal + GRe (20 mg/mL D-gal + 50 μg/mL GRe); BF, bright field; GRe, Ginsenoside Re.

Oxidative stress is a major cause of oocyte aging that often leads to mitochondrial dysfunction and developmental decline. To assess intracellular oxidative status, ROS levels were measured by H_2_DCFDA fluorescence staining. ROS levels were significantly increased in the control and aged-like condition groups (*p* < 0.01), demonstrating the establishment of an oxidative stress model. However, compared with the aged-like condition group, ROS levels were significantly decreased in the D-gal + GRe group (*p* < 0.01) (Figure 2A,B). These results suggest GRe may have an antioxidant effect. Moreover, the (Figure S1) results indicate the antioxidant capacity of alone-GRe treatment on oocytes during maturation as compared to the other groups. These results confirmed that GRe has a direct protective effect on oocytes, rather than neutralizing D-gal in the medium.

### 2.3. Effects of Ginsenoside Re on Mitochondrial Function

**Figure 3:**
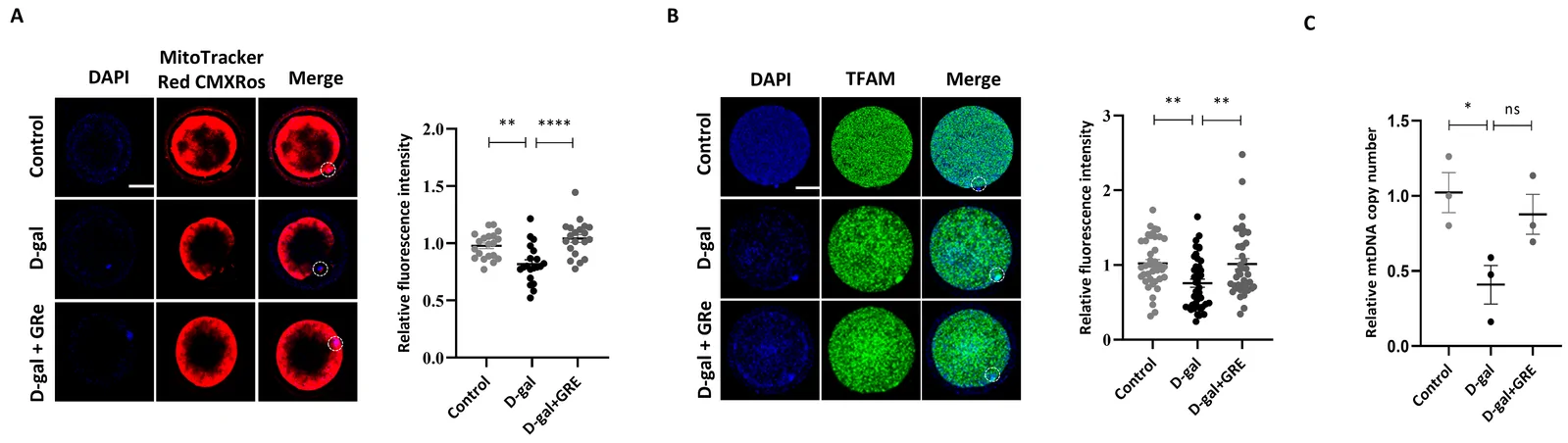
Effects of GRe treatment on mitochondrial activity and mtDNA copy number. (**A**) Representative images of mitochondrial membrane potential (MitoTracker Red CMXRos). Original magnification: ×200. Quantification of fluorescence intensity (n = 20). (**B**) Representative images of TFAM. Original magnification: x200. Quantification of fluorescence intensity (n = 40). (**C**) Relative copy number of mitochondrial DNA. All experiments were repeated three times, and the data are presented as mean ± SE. ns = not significant; * *p* < 0.05; ** *p* < 0.01; **** *p* < 0.0001. D-gal, (20 mg/mL D-gal); D-gal + GRe (20 mg/mL D-gal + 50 μg/mL GRe); DAPI, 4′,6-diamidino-2-phenylindole; *TFAM*, Mitochondrial Transcription Factor A; GRe, Ginsenoside Re.

Mitochondrial dysfunction is a hallmark of oocyte aging and affects energy production, cellular homeostasis, and mitochondrial genome maintenance. To assess the mitochondrial function, MitoTracker Red CMXRos staining, TFAM immunofluorescence, and mtDNA copy number analyses were performed. MitoTracker Red CMXRos staining revealed a significant reduction in the fluorescence intensity in aged oocytes compared with that in the control group (*p* < 0.01), indicating a loss of mitochondrial membrane potential. Treatment with GRe significantly improved mitochondrial membrane potential relative to that in the aged-like condition group (*p* < 0.0001), although the ratio remained slightly lower than that observed in the control group (Figure 3A). Immunofluorescence analysis of TFAM demonstrated decreased expression in D-gal-treated oocytes (*p* < 0.0001), consistent with impaired mitochondrial transcriptional activity. GRe treatment significantly upregulated TFAM expression compared with that in the aged group (*p* < 0.01), suggesting partial restoration of mitochondrial biogenesis (Figure 3B). Quantification of mtDNA copy number showed a significant decline in aged oocytes compared with the control group (*p* < 0.05). Although GRe treatment increased mtDNA levels, the difference was not statistically significant compared with the D-gal-treated group (Figure 3C). Collectively, these findings indicate that GRe enhances mitochondrial function in D-gal-treated oocytes by improving membrane potential and preserving mitochondrial integrity.

### 2.4. Effects of Ginsenoside Re on Lipid Droplets and Mitochondrial Mass

**Figure 4:**
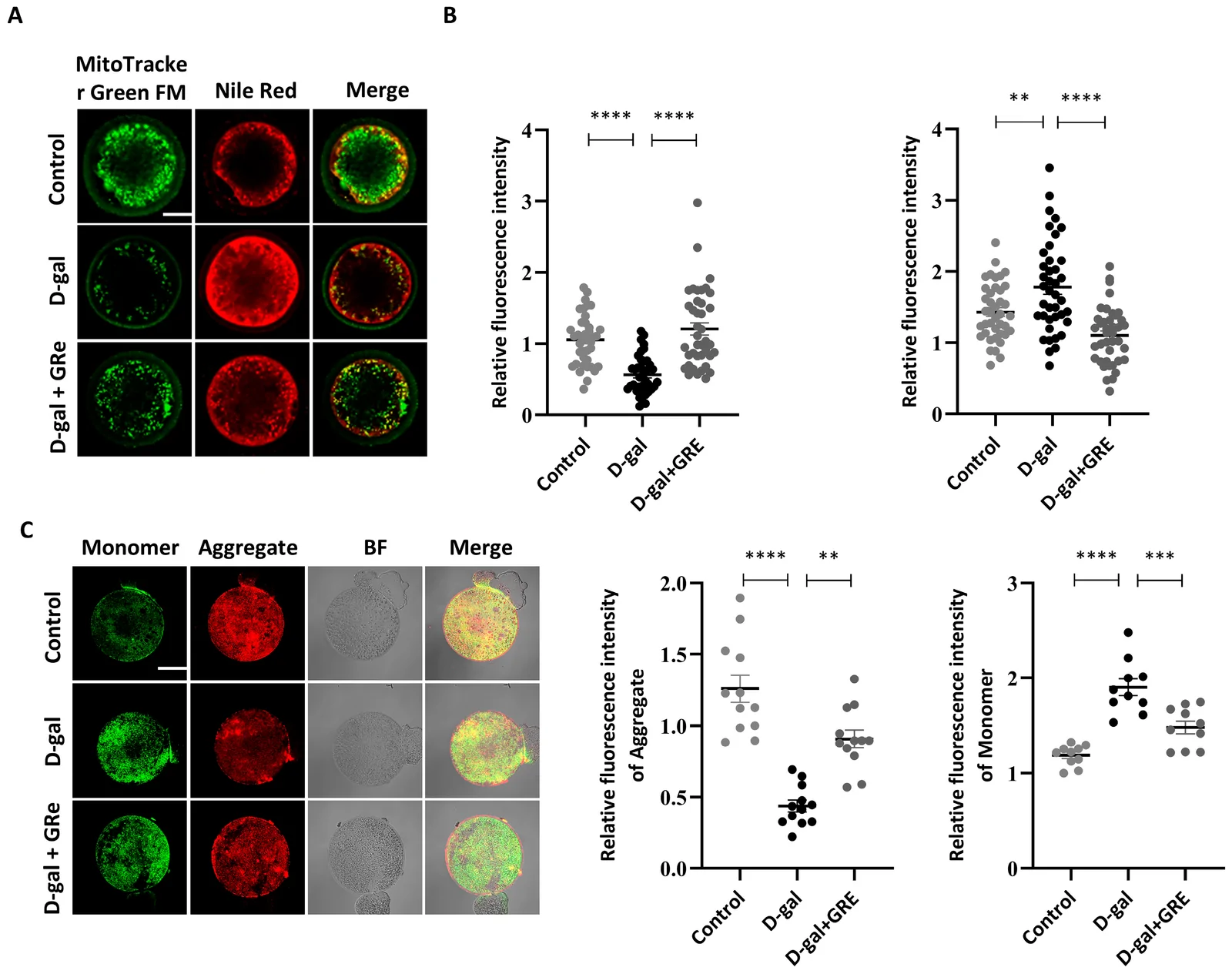
Effects of GRe treatment on mitochondrial distribution and intracellular lipid. (**A**) Representative images of mitochondrial distribution (MitoTracker Green FM) and lipid droplets (Nile Red). Original magnification: ×400. (**B**) Quantification of MitoTracker Green FM intensity (n = 40). (**C**) Quantification of mitochondria membrane potential via JC-1 staining (n = 40). All experiments were repeated three times, and data are presented as mean ± SE. ** *p* < 0.01; *** *p* < 0.001; **** *p* < 0.0001. D-gal, (20 mg/mL D-gal); D-gal + GRe (20 mg/mL D-gal + 50 μg/mL GRe); GRe, Ginsenoside Re.

In order to assess the impact of D-gal-induced aging-like conditions and GRe treatment on oocyte lipid and mitochondria, we measured intracellular lipid accumulation (lipid droplets) and mitochondrial activity in the control, aged-like condition, and D-gal + GRe groups. MitoTracker Green FM staining results indicated that aged oocytes exhibited an aberrant and clustered mitochondrial distribution pattern (*p* < 0.0001), while the GRe-treated group demonstrated a more uniform mitochondrial distribution akin to that of the control group (*p* < 0.0001) (Figure 4A). In addition, Nile Red staining showed that lipid droplet accumulation increased in aged oocytes (*p* < 0.01), which was significantly reduced after GRe treatment (*p* < 0.0001) (Figure 4B). Furthermore, we used JC-1 staining to check the effects of lipid droplet accumulation on mitochondrial membrane potential (ΔΨ_m_). The mitochondrial aggregates were significantly higher in the control and GRe-treated oocytes as compared to the D-gal group (Figure 4C). These results suggest that GRe prevents excessive lipid accumulation in aged oocytes, thereby helping maintain cellular metabolic integrity and oocyte quality.

### 2.5. Ginsenoside Reactivates the SIRT1/Nrf2 Signaling Pathway

**Figure 5:**
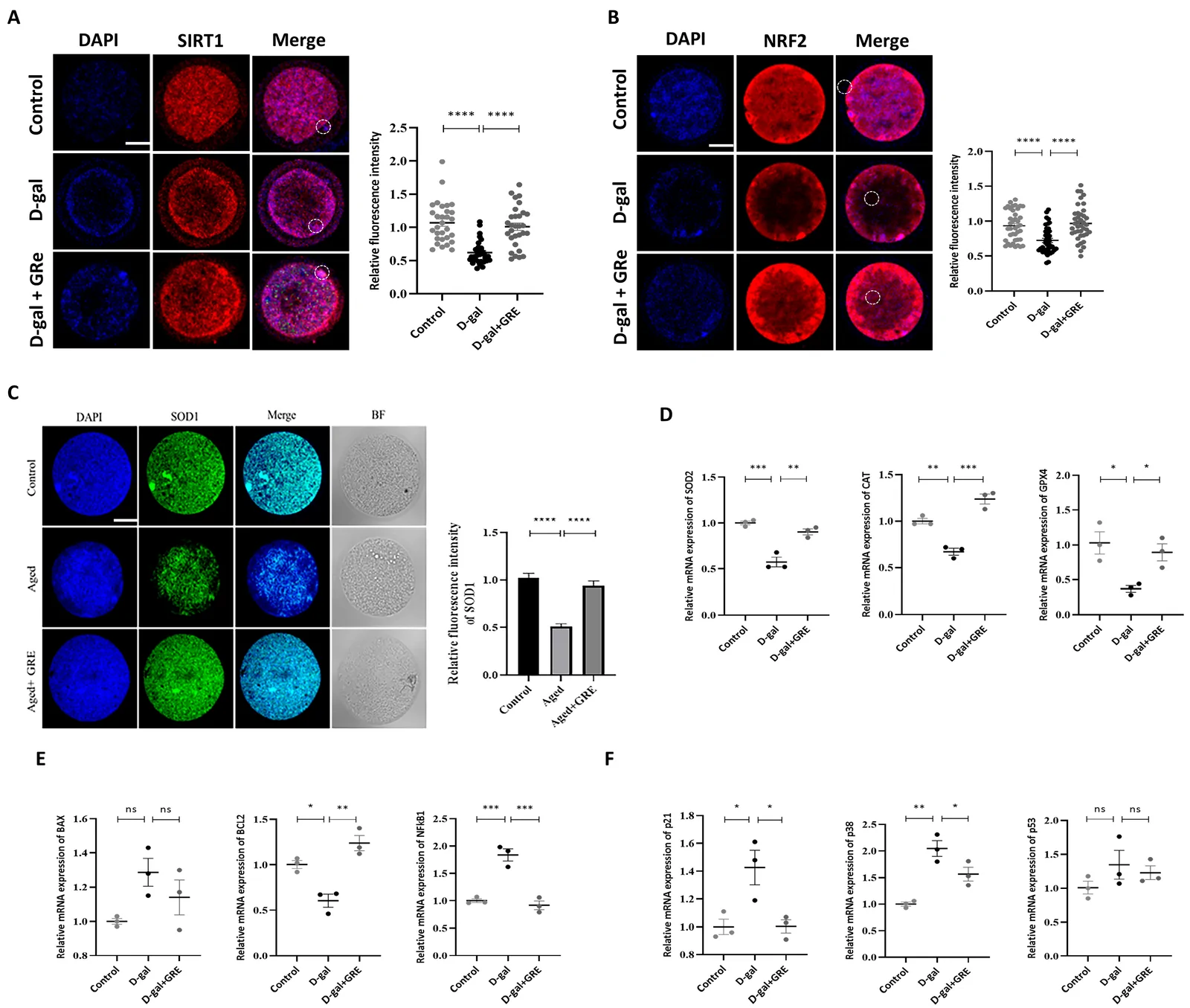
Effects of GRe treatment on the expression of genes related to the SIRT1/Nrf2 signaling pathway. (**A**) Representative images of SIRT1 immunofluorescence. Original magnification: ×200. Quantification of signal intensity and expression of SIRT1 (n = 40). (**B**) Representative images of NRF2 immunofluorescence. Original magnification: x200. Quantification of signal intensity and expression of Nrf2 (n = 30). (**C**) Immunofluorescence images of SOD1 in control, D-gal, and D-gal + GRe-treated MII stage oocytes (n = 40). (**D**) Relative mRNA expression of SOD2, CAT, and GPX4 was analyzed in control, D-gal, and D-gal + GRe-treated MII-stage bovine oocytes. (**E**) Relative mRNA expression levels of genes involved in mitochondrial functioning and stress, like BAX, BCL2, and NF-κB. (**F**) Relative mRNA expression levels of genes involved in mitochondrial dysfunction and apoptosis, like p21, p38, and p53. All experiments were repeated three times, and the data are presented as mean ± SE. ns = not significant; * *p* < 0.05; ** *p* < 0.01; *** *p* < 0.001; **** *p* < 0.0001. Aged-like condition, 20 mg/mL D-gal; D-gal + GRe, 20 mg/mL D-gal + 50 μg/mL GRe; DAPI, 4′,6-diamidino-2-phenylindole; *SIRT1*, Sirtuin 1; *Nrf2*, Nuclear factor erythroid 2-related factor 2; *SOD1*, Superoxide dismutase 1; *SOD2*, Superoxide dismutase 2; *CAT*, Catalase; *GPX4*, Glutathione peroxidase 4; *p21*, Cyclin-dependent kinase inhibitor 1A; *p38*, Mitogen-activated protein kinase 14; *NFkB1*, Nuclear factor kappa-light-chain-enhancer of activated B cells; *p53*, Tumor protein p53; *BAX*, BCL2-associated X; *BCL2*, B-cell lymphoma 2.

To elucidate the molecular mechanism underlying GRe’s protective effects against oocyte aging, gene expression in the SIRT1/Nrf2 signaling pathway was assessed by immunofluorescence and RT-qPCR. SIRT1 and Nrf2 protein levels were higher in the D-gal + GRe group than in the aged-like condition group (*p* < 0.0001) (Figure 5A,B). For additional confirmation, we analyzed the protein level of Sirt1 and NRF2 via Western blot. The results indicate that SIRT1 and Nrf2 protein levels were higher in the D-gal + GRe group than in the aged-like condition and control group (*p* < 0.0001), and actin was used as the loading control (Figure S1A). Furthermore, the SIRT1 downstream target SOD1 was analyzed via immunofluorescence, and the results showed a significant reduction (*p* < 0.0001) in SOD1 protein levels in D-gal-treated oocytes (Figure 5C). However, GRe treatment significantly saved the oocyte SOD1 protein level. Moreover, mRNA expression levels of SIRT1 downstream antioxidant target genes, including *SOD2*, *CAT*, and *GPX4*, were analyzed. Compared with the control group, the expression of all target genes was significantly decreased in the aged-like condition group (*p* < 0.05, *p* < 0.01, *p* < 0.001) (Figure 5D). This suggests dysfunction of the antioxidant defense system. Importantly, GRe treatment restored the expression levels of these genes to control levels or even increased them above control levels, suggesting activation of the antioxidant signaling pathway. In addition, the expression of inflammation- and senescence-related genes, except for *p53*, was significantly upregulated in the aged-like condition group (*p* < 0.05, *p* < 0.01, *p* < 0.001) but was significantly downregulated after GRe treatment (*p* < 0.05, *p* < 0.001) (Figure 5E). Moreover, the expression level of the pro-apoptotic gene *BAX* did not differ significantly between the groups; however, its increased expression in the aged-like condition group tended to decrease after GRe treatment in the D-gal + GRe group. In contrast, the anti-apoptotic gene *BCL2* was significantly upregulated in the GRe-treated group (*p* < 0.05) compared with that in the aged-like condition group (*p* < 0.01) (Figure 5F). These results suggest that GRe contributes to protection and redox homeostasis in D-gal-treated oocytes, primarily by activating the SIRT1/Nrf2 signaling pathway and regulating inflammation- and apoptosis-related gene expression.

### 2.6. Molecular Analysis of Ginsenoside Re at the Allosteric Site of bSIRT1

**Figure 6:**
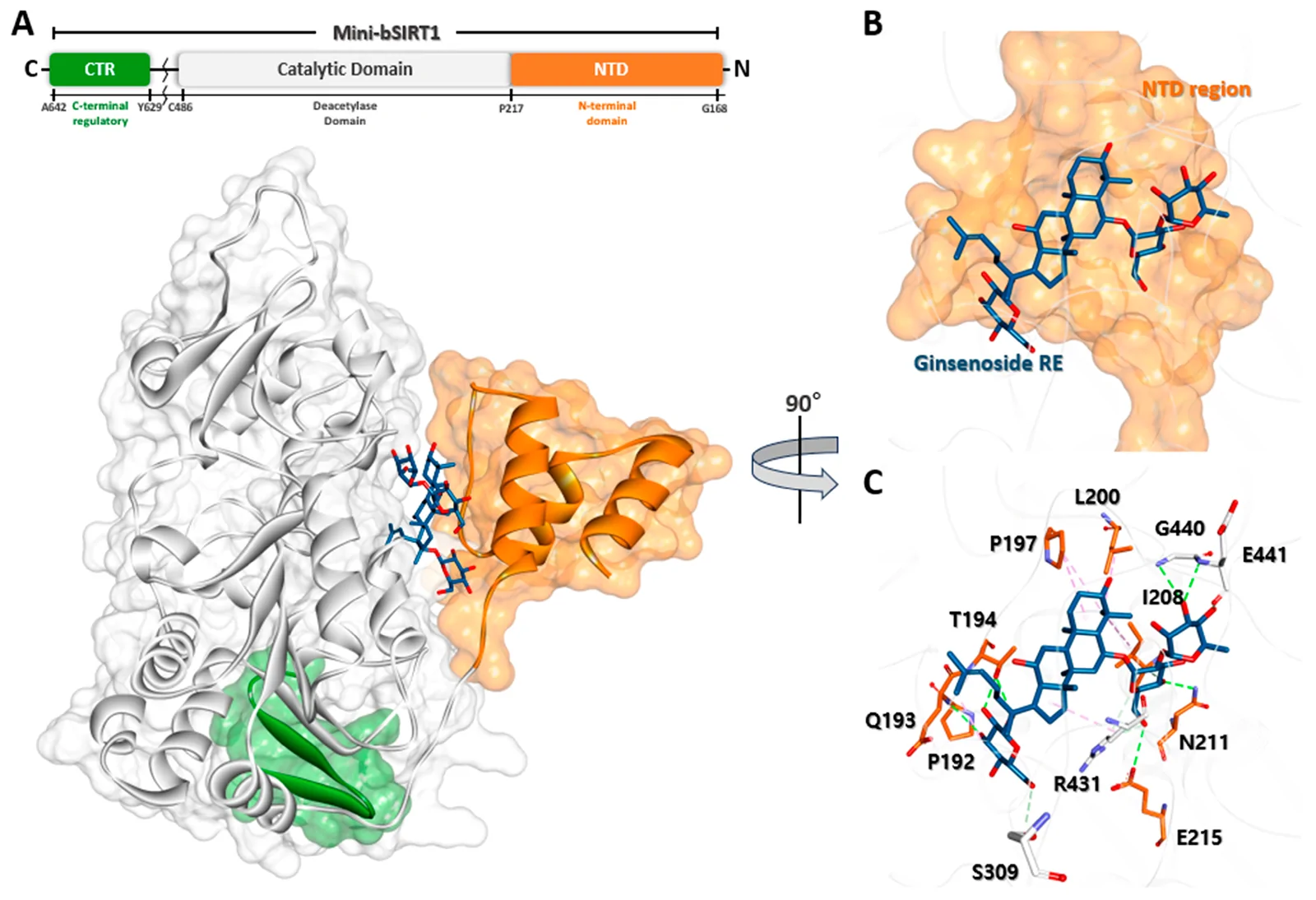
Structural and conformational analysis of bSIRT1 with GRe. (**A**) Structure of the mini-bSIRT1 and GRe complex. The deacetylase domain is colored in white, and the N-terminal domain (NTD) is colored in orange. GRe is represented as a blue stick model at the interface between the two domains. (**B**) Surface representation showing GRe positioned at the allosteric binding pocket of the NTD. (**C**) Detailed interactions of GRe with residues in the catalytic and allosteric domains. Residues from the catalytic domain and the NTD are depicted as white and orange sticks, respectively. Hydrogen bonds are indicated by green dashed lines, and hydrophobic/alkyl interactions are represented by pink dashed lines.

The bSIRT1 model predicted the mini-SIRT1 form, which includes the deacetylase catalytic core and the NTD (Figure 6A). The mini-SIRT1 construct has been widely employed in structural and functional studies because the full-length protein contains highly disordered termini that are difficult to resolve experimentally [34]. Given the bulky triterpenoid scaffold and multiple glycosidic substituents of GRe, binding within the narrow catalytic channel is sterically unfavorable. Instead, molecular docking was performed at the NTD allosteric site, which is known to bind small molecules such as STACs [35,36]. Among the 20 docking runs, 16 poses clustered within an RMSD of 2.5 Å, indicating a highly consistent docked conformation. The representative binding mode from the dominant cluster revealed that the GRe was located at the allosteric binding pocket of the NTD (Figure 6B,C). Alkyl hydrophobic interactions were established with P197, L200, and I208, which stabilized the steroidal scaffold within the hydrophobic pocket. Several hydroxyl groups of the glycosidic substituents extended toward the pocket surface, forming hydrogen bonds with N211 and E215, whereas the terminal sugar moieties engaged in additional polar interactions with P192, Q193, and T194 at the pocket entrance. Importantly, N215 and E215 are key residues for interaction with STACs, where site-directed mutations abolish their activating effects [35]. Consistent with this, T194, P197, L200, and I208 in bSIRT1 correspond to residues that have been experimentally validated as critical for STAC binding in hSIRT1 [35]. This correspondence further underscores the functional relevance of the observed GRe binding mode. Beyond allosteric sites, GRe interacted with residues in the catalytic region. Hydroxyl groups from the sugar moieties established hydrogen bonds with G440 and E441. In contrast, the dammarane backbone formed hydrophobic interactions with R431 and S309. By interacting with residues located at both the allosteric and catalytic sites, GRe was likely to stabilize the closed conformation required for the catalytic activation of SIRT1 [35]. Collectively, these results indicate that GRe engages both the NTD and catalytic regions through complementary hydrophobic and hydrogen bond interactions, supporting its potential role as an allosteric activator of SIRT1.

### 2.7. Ginsenoside Re Improves Embryo Developmental Potential

To evaluate the effects of GRe on embryonic developmental competence, embryo development was assessed after IVF (Table 1). Embryos derived from D-gal-treated oocytes showed significantly reduced cleavage rates at 48 h and blastocyst formation rates at day 8 compared with the control group. However, GRe treatment during oocyte maturation restored developmental competence. The D-gal + GRe group showed a significantly higher blastocyst development rate than that of the aged-like condition group (*p* < 0.05), suggesting that GRe plays a key role in alleviating the detrimental effects of oocyte aging on embryonic development.

## 3. Discussion

The trend of delayed childbearing over the past 30 years has increased the risk of infertility [37]. Studies on artificial insemination using donor sperm have shown a significant decrease in pregnancy rates in oocytes retrieved from women aged over 35 years [38]. In addition, IVF success rates have been shown to decrease significantly after the age of 35 years [39].

The deterioration of oocyte quality is increasingly recognized as an important cause [40] and is closely related to the functional decline of aged oocytes. Mitochondrial dysfunction is a major cause of oocyte aging [41]. Several therapeutic strategies targeting antioxidants or the SIRT1/Nrf2 signaling pathway have been studied [42,43].

GRe is the main bioactive component of ginseng and has superior biological activity compared with other ginsenosides [44]. In addition, GRe exerts antioxidant effects in various cell types and alleviates mitochondrial dysfunction in SH-SY5Y cells [29,45]. Moreover, it has been classified as a major ginsenoside that increases the expression of *SIRT1* [46,47]. However, the effects of GRe on oocyte aging are poorly understood. Therefore, this study was designed to investigate the effects of GRe treatment on mitochondrial function and on the activation of the SIRT1/Nrf2 signaling pathway under aging-like conditions in bovine oocytes.

In order to create oocyte aging-like conditions, we directly treated cumulus–oocyte complexes (COCs) in IVM medium with 20 mg/mL D-galactose. Few studies have directly applied D-galactose supplementation to culture media to cause oocyte aging in vitro [48], despite the fact that several have reported on reproductive aging models utilizing D-galactose injection in rodents [47,48]. As a result, the concentration used in this study was chosen based on earlier cell-aging models that made extensive use of D-gal [49,50]. Using this model, GRe significantly improved oocyte maturation at 50 µg/mL in a dose-dependent manner, whereas a higher concentration (100 µg/mL) decreased the maturation rate, consistent with previous reports of toxicity at excessive doses. Furthermore, decreased SIRT1 expression, a key regulator of cellular aging, together with increased p21 and p53 expression, confirmed the aging effects of D-gal and supported the protective role of GRe. Collectively, these findings suggest that GRe can attenuate D-gal-induced oocyte aging, although further studies are required to optimize GRe concentrations for oocyte culture systems. It is well established that oocyte quality determines the embryo’s developmental potential after fertilization [51]. Therefore, we evaluated blastocyst formation after in vitro fertilization. Oocytes treated with GRe during IVM showed significantly improved blastocyst development rates.

Aged oocytes have decreased fidelity of protective mechanisms against ROS [52,53]. During biological metabolic processes, an increase in ROS generated by mitochondria in oocytes causes spontaneous mitochondrial damage [5]. Mitochondrial dysfunction causes a decrease in mitochondrial membrane potential [54], mtDNA copy number, mitochondrial distribution, and an increase in lipid peroxidation. TFAM is a key protein that regulates the replication, transcription, stabilization, and packaging of mtDNA and is closely related to mitochondrial dysfunction. Previous studies have shown that suppression of TFAM expression in zebrafish embryos reduced mtDNA copy number and mitochondrial ATP production [55], whereas overexpression of TFAM increased mtDNA copy number and mitochondrial complex enzyme activity [56]. Our results suggest that GRe affects mitochondrial function recovery and regulation of energy homeostasis.

The SIRT1/Nrf2 signaling pathway plays a key role in antioxidant defense, cell survival, and mitochondrial homeostasis [57,58]. SIRT1 is widely recognized as an important regulator of cellular stress responses and mitochondrial homeostasis [59], and a decline in SIRT1 activity has been associated with aging-related dysfunction in mammalian oocytes [60]. Reduced SIRT1 expression has been linked to elevated ROS levels, impaired mitochondrial function, and decreased developmental competence in aged oocytes [19,61,62]. Moreover, SIRT1 directly deacetylates and stabilizes Nrf2 [63,64], suggesting that reduced SIRT1 activity may weaken Nrf2-mediated antioxidant defense and thereby exacerbate oxidative damage during oocyte aging.

The modulation of antioxidant downstream target genes associated with the SIRT1/Nrf2 signaling cascade, together with the observed alterations in genes linked to cellular aging, inflammation, and apoptosis, collectively suggests that GRe exerts a stimulatory effect on this pathway. This interpretation is further corroborated by the upregulated expression of the central regulatory proteins SIRT1 and Nrf2. Also, molecular docking results showed that GRe could bind to the allosteric site of bSIRT1, suggesting the potential for increased protein activity. In addition, our results suggested that GRe may mediate ROS reduction and mitochondrial function enhancement through this pathway. To our knowledge, this is the first study to elucidate the mechanisms underlying GRe effects in bovine oocytes.

In conclusion, supplementation of GRe in IVM medium neutralizes aging-like oxidative stress (D-gal-induced) during oocyte maturation by promoting mitochondrial health through activation of the SIRT1/Nrf2 signaling pathway in mature oocytes.

## 4. Materials and Methods

### 4.1. Chemicals

All chemicals and reagents not otherwise specified were purchased from Merck (Sigma-Aldrich, St. Louis, MO, USA).

### 4.2. Ethics

The animal study protocol was approved by the Animal Care Facility of the Gyeongsang National University Institute of Animal Care Committee (GNU 230425-A0088). None of the animals were used directly in this study, and all bovine ovaries were collected from a local slaughterhouse.

### 4.3. Experimental Design

To initiate our experiment, we collected cumulus–oocyte complexes (COCs) from slaughterhouse bovine ovaries. Increasing concentrations of GRe were treated to the COCs for 24 h to check the effect of GRe on oocyte in vitro maturation. Next, the COCs were randomly divided into three groups: control, D-galactose (D-gal) treated (20 mg/mL), and D-gal + GRe (25 μg/mL, 50 μg/mL, and 100 μg/mL). After careful analysis, we found that GRe at 50 μg/mL significantly neutralizes D-gal-induced toxicity (the basic criterion was polar body extrusion).

Futhermore, after finalizing the dose of GRe, the reactive oxygen species (ROS) were analyzed in the control, D-gal (20 mg/mL)-, and D-gal + Gre (50 μg/mL)-treated samples (MII-stage oocytes). Next, the mitochondrial analysis was performed via MitoTracker Red (Cat. No. M7512) (Waltham, MA, USA). Furthermore, TFAM protein and mtDNA copy number were also analyzed in the control, D-gal (20 mg/mL)-, and D-gal + GRe (50 μg/mL)-treated samples. In addition, mitochondrial and lipid content was analyzed using MitoTracker Green (Waltham, MA, USA) and Nile Red staining. Moreover, mitochondrial membrane potential (Δψm) and mitochondrial mass were assessed in control, D-gal (20 mg/mL), and D-gal + GRe (50 μg/mL) oocytes using JC-1 and Mito tracker green staining.

SIRT1 and NRF2 proteins play significant roles in mitochondrial function, so we observed the protein levels of in vitro mature oocytes from the control, D-gal (20 mg/mL), and D-gal + GRe (50 μg/mL) groups. Next, we examined the gene expressions of SIRT1, NRF2, SOD1, P21, and p-38 in the control, D-gal, and D-gal + GRe conditions in in vitro-matured bovine oocytes. Furthermore, the gene expression levels of BAX, SOD2, CAT, GPX4, NF-KB, p53, and BCL2 were examined in control, D-gal, and D-gal + GRe conditions in vitro-matured bovine oocytes.

Lastly, we examined the interaction of GRe with bovine SIRT1 protein and verified that GRe-mediated SIRT1 activation can activate NRF2 signaling, thereby improving mitochondrial function and resisting D-gal-induced aging-like oxidative stress conditions.

### 4.4. Collection of Bovine Cumulus–Oocyte Complex

Ovaries were collected from a slaughterhouse in Gimhae, immersed in 0.9% NaCl solution at 37.5 °C, and transferred to the laboratory within 1 h. After washing ovaries with saline, cumulus–oocyte complexes (COCs) were retrieved from ovarian follicles (2–8 mm in diameter) using an 18-gauge needle connected to a 10-mL disposable syringe. Aspirated follicle fluid was discharged into the 50-mL tube containing Tyrode lactate-HEPES [TL-HEPES; calcium chloride 2 mM (Cat. No. C-7902), HEPES 10 mM (H-6147), 100 IU/mL penicillin, phenol red 1 μL/mL (P-0290), potassium chloride 3.2 mM (P-5405), sodium bicarbonate 2 mM (S-5761), sodium biphosphate 0.34 mM (S-5011), sodium chloride 114 mM (S-5886), sodium lactate 10 mM (L-4263), and 0.1 mg/mL streptomycin] solution at 38.5 °C, and oocytes were collected using a stereomicroscope. After washing with TL-HEPES, COCs with three layers of densely compacted cumulus cells were selected.

### 4.5. In Vitro Maturation

COCs were washed six times with in vitro maturation (IVM) medium [Tissue Culture Medium-199 (TCM199; Cat. No. 11,150-059) supplemented with cysteine 0.6 mM, epidermal growth factor 10 ng/mL (EGF, E-4127), 10% (*v*/*v*) fetal bovine serum (16,000-044), follicle-stimulating hormone 10 μg/mL (HOR-285), 1 μg/mL estradiol-17β, and sodium pyruvate 0.2 mM (P-5280)], and COCs, typically numbering 30 to 35 per well, were cultured in 500 µL of IVM medium and incubated at 38.5 °C in a controlled humidified atmosphere containing 5% CO_2_ for 22 h.

### 4.6. In Vitro Oocyte Aging

To induce aging-like oxidative stress conditions in bovine oocytes, COCs were cultured in IVM medium supplemented with 20 mg/mL D-galactose (D-gal; Cat. No. G0750) for 22 h. This model was established by inducing oxidative stress through the accumulation of intracellular reactive oxygen species (ROS) in the cultured cells. Based on the preliminary evaluation of oocyte maturation rate and subsequent assessment of aging-related characteristics, including ROS levels and mitochondrial membrane potential (JC-1), 20 mg/mL D-gal was selected for all subsequent experiments.

### 4.7. Ginsenoside Re Treatment

GRe (Cat. No. 77960) was dissolved in dimethyl sulfoxide (DMSO) and co-administered with 20 mg/mL D-gal in IVM medium. The final concentrations of GRe were adjusted to 25, 50, and 100 μg/mL following dilution, with the final concentration of DMSO maintained below 0.5% in all groups.

### 4.8. Assessment of Oocyte Maturation

After IVM, COCs were treated with 0.1% hyaluronidase and gently pipetted to remove cumulus cells. Denuded oocytes were observed under a stereomicroscope. The presence of the first polar body (PB1) was used to assess oocyte maturation, and the maturation rate was calculated as the percentage of PB1-extruded oocytes over the total number of oocytes examined.

### 4.9. Reactive Oxygen Species (ROS) Assay

Intracellular ROS levels were evaluated using 2′,7′-dichlorodihydrofluorescein diacetate (H_2_DCFDA; Cat. No. D6883). Oocytes were incubated in phosphate-buffered saline (PBS) containing 10 μM H_2_DCFDA at 38.5 °C in the dark for 30 min. Following washing, oocytes were mounted on glass slides, and fluorescence images were captured using an Olympus IX71 microscope. Fluorescence intensity was quantified using the ImageJ software (version number ij154) (National Institutes of Health, Bethesda, MD, USA; https://imagej.net/ij/download.html accessed on 1 July 2026).

### 4.10. MitoTracker Red CMXRos Staining

The mitochondrial membrane potential was determined using MitoTracker Red CMXRos (Cat. No. M7512). Denuded oocytes were incubated in 500 nM MitoTracker Red CMXRos solution at 38.5 °C in the dark for 30 min. After washing, oocytes were mounted on glass slides for imaging. Fluorescence was captured using a laser-scanning confocal microscope (Fluoview FV 1000; Olympus, Tokyo, Japan). Fluorescence intensity was quantified using the ImageJ software.

### 4.11. JC-1 and Nile Red Staining

JC-1 (Sigma Aldrich (St. Louis, MO, USA), Cat. No. T3168) was used to label mitochondria [65]. Oocytes were incubated in 2.0 μg/mL at 38.5 °C in the dark for 25 min. The samples were washed thrice with D-PBS and mitochondrial analysis was performed using a confocal laser-scanning microscope (Fluoview FV 1000, Olympus, Japan). For staining lipid droplets, Nile Red (Cat. No. 72485) was used at 3.14 μM under the same conditions. After staining, oocytes were washed with PBS and imaged using a fluorescence microscope. Fluorescence intensity and subcellular distribution were analyzed using the ImageJ software.

### 4.12. Mitochondrial DNA (mtDNA) Copy Number Analysis

A total of 15–20 MII-stage oocytes per group were collected, and mtDNA was extracted using 20 μL of lysis buffer (20 mM Tris, 0.4 mg/mL proteinase K, 0.9% Nonidet-40, and 0.9% Tween 20) at 55 °C for 30 min and subsequently at 95 °C for 5 min. The SsoAdvanced™ Universal SYBR Green mix (Bio-Rad, Hercules, CA, USA) was used for real-time polymerase chain reaction (PCR), which involved an initial denaturation at 95 °C for 5 min, followed by 44 cycles at 95 °C for 10 s and at 60 °C for 30 s. SYBR Green fluorescence was measured at the end of each extension step. Fluorescence quantification was performed to detect the mitochondrial gene *COX1*. Simultaneously, the expression levels of the nuclear single-copy gene *NTHL1* were measured. To determine mt DNA copy number, the relative concentrations of *COX1* and *NTHL1* were calculated. Specific primer sequences (Table 2) were designed using the National Center of Biotechnology Information (NCBI) database. The relative mtDNA copy number (N) was calculated using the following formula: $N\;=\;2^{-\left(Ct_{mt}-Ct_{n}\right)}$, where the Ct_mt_ value of the mitochondrial gene and Ct_n_ is the Ct value of the nuclear single-copy gene.

### 4.13. Immunofluorescence

To determine the expression levels of SIRT1, Nrf2, and Mitochondrial Transcription Factor A (TFAM), MII-stage oocytes were fixed with 4% paraformaldehyde for 30 min, permeabilized with 0.5% Triton X-100 for 30 min, and blocked with 1% bovine serum albumin (BSA) for 1 h [66]. The oocytes were incubated overnight at 4 °C with anti-SIRT1 (Cat. No. sc-74504), anti-Nrf2 (orb11165), and anti-TFAM (PA-568789) primary antibodies, followed by incubation with an Alexa Fluor 568-conjugated anti-mouse (A-10037), Alexa Fluor 594-conjugated anti-rabbit (A-21207), and Alexa Fluor 488-conjugated anti-rabbit (A-21206) secondary antibodies for 90 min at 25 °C. Nuclei were counterstained with DAPI for 5 min. Fluorescent signals were observed using a laser-scanning confocal microscope (FV1000; Olympus, Tokyo, Japan).

### 4.14. In Vitro Fertilization and In Vitro Culture

After IVM, frozen semen straws were thawed in a water bath at 37.5 °C for 1 min, and sperm were washed with Dulbecco’s phosphate-buffered saline (DPBS). The sperm suspension was centrifuged at 750× *g*, 25 °C for 5 min. The sperm pellet was resuspended in 500 µL heparin solution (20 µg/mL) and subsequently diluted with 4.5 mL of in vitro fertilization (IVF) medium [penicillin (100 IU/mL), sodium pyruvate (22 µg/mL), streptomycin (0.1 mg/mL), and BSA (6 mg/mL), added to TL-HEPES solution]. The sperm suspension was incubated at 38.5 °C in a humidified atmosphere containing 5% CO_2_ for 15 min. The final sperm concentration was adjusted to 1 × 10^6^ sperm/mL. Matured COCs were co-incubated with the prepared sperm suspension in 500 µL IVF medium at 38.5 °C in a humidified atmosphere containing 5% CO_2_ for 20 h. After IVF, cumulus cells were removed from presumptive zygotes by repeated pipetting. The zygotes were then washed with 500 µL of synthetic oviductal fluid (SOF) medium [5 ng/mL ITS (insulin, transferrin, sodium selenite, Cat. No. 11074547001), 100 ng/mL EGF, and 4 mg/mL BSA]. Embryos were cultured in 4-well dishes at 38.5 °C in a humidified atmosphere containing 5% CO_2_, 5% O_2_, and 90% N_2_. Embryo development was evaluated under a stereomicroscope at 48 h (cleavage) and day 8 (blastocyst formation) post-insemination.

### 4.15. RNA Isolation and Quantitative Real-Time PCR (RT-qPCR)

The expression levels of genes involved in the SIRT1/Nrf2 signaling pathway, inflammation, senescence, and apoptosis were analyzed by quantitative real-time PCR (qRT-PCR). Bovine mature oocytes were used to collect mRNA as previously described [67]. In short, Dynabeads mRNA direct kit was used, and the manufacturer’s instructions (Dynal AS, Oslo, Norway) were followed. A minimum of fourty oocytes per biological sample was used to isolate the mRNAs. The purified mRNA concentration was determined using NanoDrop 2000c at 260 nm. To convert mRNA to complementary DNA (cDNA), Superscript III reverse transcriptase (Bio-Rad Laboratories, Hercules, CA, USA, cat. #1708891) was used. The expression levels of these genes were normalized to that of glyceraldehyde-3-phosphate dehydrogenase (*GAPDH*). Primers were designed from the mRNA sequences of the genes and purchased from Macrogen (Seoul, Republic of Korea). qRT-PCR was performed using the CFX98 system (Bio-Rad Laboratories) with 1× iQ SYBR Green Supermix (iQ™ SYBR^®^ 327 Green 328 Supermix kit, Bio-Rad Laboratories) and 3 µL of diluted cDNA as template. All cDNA samples were subjected to qRT-PCR to detect variations in the expression of other genes using *GAPDH* primers. After confirming that *GAPDH* expression was not significantly different between the samples, all transcripts were quantified using qRT-PCR. There were 35 amplification cycles and the cycling conditions were as follows: 95 °C for 3 min, 95 °C for 15 s, 62 °C for 20 s, 72 °C for 30 s, and final extension for 5 min. Quantitative analysis was performed using the ^ΔΔ^Ct method. The results are reported as values relative to the mean of the endogenous control, GAPDH, after normalization to the calibrator. Intra- and inter-assay variance coefficients were calculated for all genes using qRT-PCR. The primer sequences are listed in Table 3.

### 4.16. Western Blotting

The experiments used 150–200 mature oocytes per extract. Oocytes were rinsed with D-PBS to eliminate impurities and subsequently dissolved in iNtRON Biotechnology’s PRO-PREPTM solution. Sonication disrupted cells to enhance protein extraction, followed by centrifugation at 10,000× *g* for 25 min at 4 °C to separate soluble proteins from cellular debris. The supernatant was collected, and the Bradford assay was used to quantify protein. Equal quantities (10–20 μg) were applied to 12% SDS-PAGE gels for molecular weight separation. A PVDF membrane was used for protein analysis after electrophoresis. The membrane was treated with skim milk, incubated overnight at 4 °C with a primary antibody, washed 3 times, and then incubated for 90 min with an HRP-conjugated secondary antibody, followed by a final 3 washes. The Pierce ECL Western Blotting Substrate was used for detection, and Abcam protein ladders were used for molecular weight assessment. Optical densities of bands on X-ray images were measured in ImageJ to assess protein expression levels. Western blot analysis was performed using three independent biological replicates.

### 4.17. Structural Prediction and Preparation of Bovine SIRT1

The three-dimensional (3D) structure of bovine SIRT1 (bSIRT1) was generated using AlphaFold version 2.3.0 [68]. The mini-bSIRT1 architecture, comprising the deacetylase domain and the N-terminal domain (NTD), was constructed from the corresponding amino acid sequence of bSIRT1 (UniProt ID: F1MQB8), with reference to the human mini-SIRT1 crystal structure (PDB ID: 5BTR) [69,70]. AlphaFold was run using default parameters, including up to three refinement cycles, and an AMBER relaxation step was applied to optimize side-chain geometry and resolve steric clashes. The final model was selected based on the highest per-residue Local Distance Difference Test (pLDDT) score. The selected model was prepared by adding hydrogen atoms with protonation states assigned at pH 7.0, followed by energy minimization using the AMBER force field implemented in GROMACS [71].

### 4.18. Molecular Docking Simulation

Molecular docking was performed between mini-bSIRT1 and GRe using GOLD Suite (version 5.2.2; Cambridge Crystallographic Data Center, UK) [72]. The 3D structure of GRe was obtained from PubChem (Compound CID: 441921) [73], and geometry optimization was performed using RDKit [74] with the Merck Molecular Force Field, which provides improved accuracy for small organic compounds. Molecular docking was conducted at the allosteric site of bSIRT1, corresponding to the Sirtuin-Activating Compound (STAC) binding domain. This site is located on the N-terminal region of SIRT1 and facilitates allosteric activation by stabilizing substrate interactions [75]. The docking site was defined within a 20 Å radius centered on the key residues of the NTD. Twenty independent docking runs for GRe were performed using default parameters, and docked poses were clustered based on the root-mean-square deviation (RMSD) to identify dominant binding modes. Among these, the pose with the highest docking score within the major cluster was selected as the optimal binding mode for detailed interaction analysis.

### 4.19. Statistical Analysis

Statistical analysis was performed using one-way analysis of variance (ANOVA) in GraphPad Prism 8 software (GraphPad Software, Franklin Street, Boston, MA; www.graphpad.com), followed by multiple pairwise comparisons using Tukey’s post hoc test to assess differences between groups. mtDNA copy number assay and RT-qPCR were performed on randomly selected MII-stage oocytes (15–20 oocytes per replicate) from each group. The mean fluorescence intensity across all imaging data was quantified for each MII-stage oocyte (n = 20–40) in each group. Experiments were conducted in triplicate for data analysis. ImageJ software (version: ij154) was used to generate all histogram values. Data are expressed as the mean ± standard error of the mean (mean ± SE). * *p* < 0.05; ** *p* < 0.01; *** *p* < 0.001; **** *p* < 0.0001 indicate a significant difference.

## Figures and Tables

**Table 1:** Effect of GRe treatment on embryo development.

Treatments	No. of Oocytes *	No. of Presumed Zygotes	No. of Embryos Cleaved (% ± SE) **	No. of Total Blastocysts (% ± SE) **	No. of Hatched Blastocysts (% ± SE) **
Control	209	200	136(68.89 ± 2.49) ^b^	61(30.34 ± 0.44) ^b^	12(19.83 ± 0.98)
D-gal	287	282	160 (57.02 ± 3.16) ^a^	53 (18.89 ± 0.72) ^a^	8 (15.14 ± 1.66)
D-gal + GRe	305	299	187 (62.43 ± 1.47) ^a,b^	86 (28.93 ± 0.93) ^b^	13 (15.16 ± 3.37)

^a,b^ Values with different superscripts in the same column are significantly different (*p* < 0.05). * Three replicates were performed. ** Data are calculated based on the total number of presumed zygotes.

**Table 2:** Primer sequences for mtDNA copy number analysis.

Genes	Forward Primer (5′ → 3′)	Reverse Primer (5′ → 3′)	Accession Number
*NTHL1*	GAAAAGCTACAGCCCCGTGAA	GGATGGTGCCTGGAGATGC	NM_001046397.2
*COX1*	CCTCAATTTTAGGAGCCATCA	CTGCTAATACAGGGAGCGAGA	YP_209207.1

**Table 3:** Primer sequences for RT-qPCR analysis.

Genes	Forward Primer (5′ → 3′)	Reverse Primer (5′ → 3′)	Accession Number
*GAPDH*	TTCAACGGCACAGTCAAGG	ACATACTCAGCACCAGCATCAC	NM_001034034.2
*SIRT1*	CAACGGTTTCCATTCGTGTG	GTTCGAGGATCTGTGCCAAT	NM_001192980.3
*Nrf2*	GCTCAGCATGATGGACTTGG	TGCTCCTTCTGTCGTTGACTG	NM_001011678.2
*SOD1*	CCATCCACTTCGAGGCAAAG	TCTCCAAACTGATGGACGTGG	NM_174615.2
*SOD2*	GTGAACAACCTCAACGTCGC	GGGTTCTCCACCACCGTTAG	NM_201527.2
*CAT*	TTTGACCCAAGCAACATGCC	TGTGAACACCATGCTCTGCT	NM_001035386.2
*GPX4*	AAGGAGGAGGGCTCGGATA	GGCTGAAAATTCGTGCATGG	NM_001346430.1
*NFkB1*	GAAGGAGATGGACCTCAGCG	TGCTGTCATAGATGGCGTCC	NM_001076409.1
*P21*	GCAAATATGGGTCTGGGAGA	AAATAGTCCAGGCCAGGATG	NM_001098958.2
*P38*	AGCTTCAGCAGATTATGCGTC	TCTTCGGCATCTGGGTCAAA	NM_001102174.1
*P53*	CTCAGTCCTCTGCCATACTA	GGATCCAGGATAAGGTGAGC	NM_174201.2
*BAX*	TGAGCAGATCATGAAGACAGG	GTAGAAAAGGGCGACAACCC	NM_173894.1
*BCL2*	CTTCTTTGAGTTCGGAGGGGT	GCCAGGAGAAATCAAACAGGG	NM_001166486.1

## Data Availability

The data presented in this study are available from the corresponding author upon reasonable request.

## Supplementary Materials

The following supporting information can be downloaded at https://www.mdpi.com/article/10.3390/ijms27177569/s1.
