# Supplementary material for: Ginsenoside Re Attenuates D-Galactose-Induced Aging-like Oxidative Stress in Cultured Bovine Oocytes via Activation of the SIRT1/Nrf2 Signaling Pathway
Source: Int J Mol Sci. 2026 Aug 24;27(17):7569. doi: 10.3390/ijms27177569 (PMC13566820; doi:10.3390/ijms27177569)
Supplement: Supplementary file 1 [file ijms-27-07569-s001.zip › ijms-4444013-supplementary.pdf]

Supplemental for Revision

Developmental capacity of GRe in oocytes during maturation and cleavage

exposure to d-gal markedly impeded maturation, yielding a rate of merely 55.24%. Even so, pretreatment with GRe partially reinstated maturation rates to 75.70%, suggesting that antioxidant supplementation before stress exposure improves oocyte resistance. Significantly, GRe treatment after d-gal-induced damage resulted in substantial improvements in maturation outcomes (78.68% versus 53.00% in the damage-only group), indicating that GRe can both prevent oxidative injury and promote recovery from pre-existing age-related dysfunction **(Table S1)**. These results underscore the protective potential of GRe in mitigating age-related reproductive decline via both preventative and restorative mechanisms.

Table S1. Analysis of GRE effect on oocyte, rather than D-gal neutralization in media.

**GRe pre-treatment, then D-gal-induced aging**			
**Parameter**	**D-gal only (20 mg/mL)**	**D-gal + GRe pre-treatment (50 µg/mL)**	**Control (no D-gal & GRe)**
No. of oocytes	105	107	110
Maturation (%)	58 (55.24 ± 0.24)^{c}	81 (75.70 ± 0.30)^{b}	94 (85.45 ± 0.44)^{a}
**D-gal-induced aging, then GRe recovery treatment**			
**Parameter**	**D-gal then control (D-gal removal only)**	**D-gal then GRe treatment (50 µg/mL)**	**Control (no D-gal & GRt)**
No. of oocytes	100	115	102
Maturation (%)	53 (53.00 ± 0.00)^{b}	90 (78.15 ± 0.26)^{a}	86 (84.31 ± 0.30)^{a}

^{a,b,c}Values with different superscripts in the same column are significantly different ($p < 0.05$). Letters are assigned in descending order of mean values.

GRe rescues aged oocyte NRF2, SIRT1, and SOD1 antioxidant regulators

The GRe treatment markedly increased the expression of endogenous antioxidant regulators in D-gal-induced aged oocytes. Compared with the D-gal-induced aged group, aged oocytes supplemented with GRe showed elevated protein levels of NRF2 and SIRT1 by western blot analysis **(Figure S1A)**, reaching levels equivalent to or above those of controls, whereas ageing alone significantly reduced the levels of both proteins. Furthermore, the immunofluorescence intensity of SOD1 **(Figure S1B)** was significantly reduced in aged oocytes but was markedly restored by GRe, with staining patterns like those observed in the control group. These results indicate that GRe mitigates age-related downregulation of the NRF2–SIRT1 axis and sustains SOD1 levels in oocytes, thereby improving their inherent antioxidant defenses during maturation.

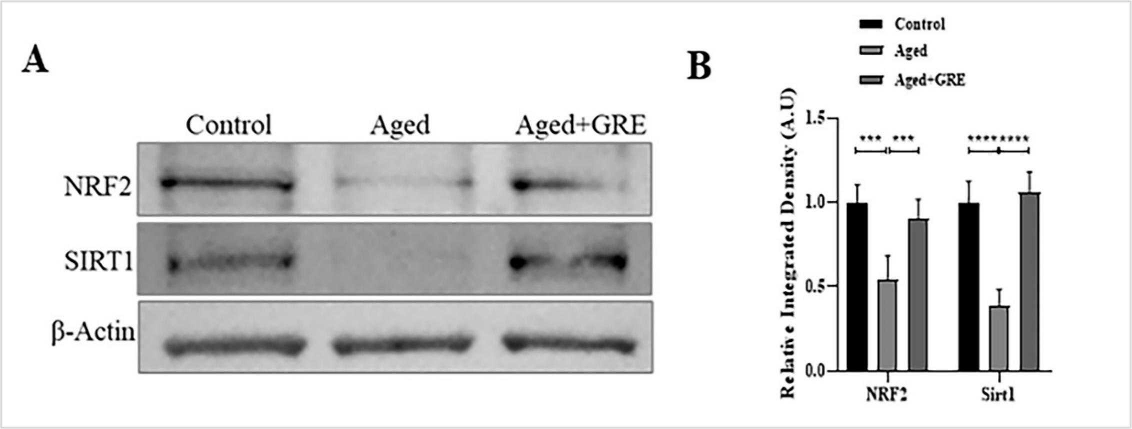

Figure S1. GRe supplementation recovers NRF2, SIRT1, and SOD1 in aged oocytes. (A) Western blot illustrations of NRF2 and SIRT1 protein expression in control, aged, and aged+GRe oocytes, with β-actin as the loading control. **(B)** Normalized relative integrated density of NRF2 and SIRT1 protein levels to β-actin. Data are presented as mean ± SEM. ***P < 0.001, **P < 0.0001.
